# Acousto−Optics: Recent Studies and Medical Applications

**DOI:** 10.3390/bios13020186

**Published:** 2023-01-25

**Authors:** Mohammadreza Omidali, Ali Mardanshahi, Mariella Särestöniemi, Zuomin Zhao, Teemu Myllylä

**Affiliations:** 1 Optoelectronics and Measurement Techniques Research Unit, Faculty of Information Technology and Electrical Engineering, University of Oulu, 90570 Oulu, Finland; 2 Research Unit of Health Sciences and Technology, Faculty of Medicine, University of Oulu, 90220 Oulu, Finland; 3 Center for Wireless Communications, University of Oulu, 90570 Oulu, Finland

**Keywords:** acousto−optics, biomedical imaging, ultrasound, biophotonics

## Abstract

Development of acousto−optic (AO) techniques has made progress in recent years across a range of medical application fields, especially in improving resolution, detection speed, and imaging depth. This paper presents a comprehensive overview of recent advancements in AO−based techniques that have been presented after the previously published review in 2017. The survey covers a description of theoretical modeling strategies and numerical simulation methods as well as recent applications in medical fields. It also provides a comparison between different techniques in terms of complexity, achieved depth in tissue, and resolution. In addition, a comparison between different numerical simulation methods will be outlined. Additionally, a number of challenges faced by AO techniques are considered, particularly in the context of realistic in vivo imaging. Finally, the paper discusses prospects of AO−based medical diagnosis methods.

## 1. Introduction

Optics and optical imaging have entered the medical research field, especially after the invention of lasers, because light is non−ionized radiation and tissue components have good optical contrast at different optical wavelengths. Thus, non−invasive optical imaging can provide physiological information about tissues. In addition, the optical properties of biological tissues are related to their molecular constituents in visible and near−infrared (NIR) spectra. Wavelength−dependent interaction with tissue allows spectroscopic imaging [1,2], which is, however, limited by high scattering and absorbance in biological tissues, preventing light from penetrating deeply. In tissue imaging, there are various optical imaging techniques, such as optical coherence tomography (OCT), which can detect ballistic and snake photons at a depth of 1 to 2 mm, and diffuse optical tomography (DOT), or functional near−infrared spectroscopy (fNIRS), which can reach a depth on the order of centimeters, but with low spatial resolution, due to the high diffusion of photons [3,4]. To overcome these limitations, another branch of optical imaging is being developed based on hybrid imaging, where the basic idea is to combine the best features of two or more measurement modalities, for instance to compensate for a low spatial resolution of a modality by using other measurement modality with a higher spatial resolution. A combination of two modalities can also provide a measurement or imaging method that is possible to realize only as a hybrid technique.

Aiming for deep tissue imaging with high contrast and spatial resolution, hybrid imaging techniques, specifically ones that combine acoustics and optics, will be most likely to succeed. At the end of the last century, photoacoustic (PA) and acoustic−optic (AO) imaging methods were introduced. Both are hybrid techniques and utilize the high resolution of ultrasound (US) imaging with photoacoustic imaging (PAI) detecting laser−generated US waves in optically absorbing components of tissue, while acousto optic imaging (AOI) detects ultrasonic−modulated optical signals from tissue. Even though the study of these two fields started at the same time, several challenges have limited AOI to ex vivo (phantom) imaging [5], whereas PAI has received considerable attention in preclinical imaging [6,7,8]. Recently, PAI has achieved great progress in preclinical applications focusing on in vivo imaging of animal brains and internal organs and determining various physiological parameters. At present, emerging clinical applications of PAI include human breast tumor/cancer imaging, prostate imaging, ophthalmic imaging, arthritis imaging, thyroid imaging, and brain functional imaging [7].

However, the progress of AOI has been relatively limited, as most work in the field is limited to theoretical aspects and sample studies. This can be explained by weak optical modulation in deep tissue and strong background light level. The basic idea of AOI involves using a spatially accurate US field to improve the spatial resolution of diffuse light. In a US modulated zone, some photons of the illuminating light will be modulated by US, shifting their frequency by the US frequency. Having thus been “tagged” with the US frequency, these photons now carry information of the US zone and can be separated from the “untagged” background photons. This results in acoustic resolution exceeding that of optical resolution in diffuse optical imaging, and allows the retrieval of local optical properties of the tissue under study [3]. AOI is sensitive both to absorption and scattering, unlike PAI, which is only sensitive to absorption. The optical contrast produced by absorption and scattering can be used to provide information about organ structure as well as tissue metabolism. As most soft−tissue components have similar acoustic impedances, US can be focused with good spatial resolution in conventional medical imaging. When light enters the focal region of a sound wave, it can be diffracted and frequency−shifted. A light detector can capture a fraction of this frequency−shifted light as it propagates out of the tissue. Moving the US focus could enable the focal region to be compared to other regions, if frequency−shifted light is filtered out from the background [9]. Although both PAI and AOI are US−mediated optical imaging techniques, their theoretical principles are different. PAI is entering clinical application, but it still meets a great challenge in human brain imaging in vivo. This is because the adult human skull strongly attenuates and distorts PA waves when they propagate out of the skull from the brain, as a result of wave mode transformation and unmatching acoustic impedances between the skull bone and the soft−tissues of the head. This disadvantage can be largely avoided by AOI, because it detects optical signals and the effect of optical parameter unmatching between bone and soft tissues is much smaller. As a result, the effect the skull has on optical signal reception is greatly reduced. Based on this viewpoint, it is hypothesized that AOI should prove more successful in non−invasive imaging and sensing of the human brain. Another advantage of AOI is that its imaging depth is potentially twice as deep as that of PAI at its current state [7]. However, in this technique, the ratio between US−modulated light (tagged) signals and unmodulated light is low, making it the main challenge for AOI [10]. As an example, [11] pointed out that the coherence length of an incident laser should be 7 cm or more for effectively detecting optical speckle signals transmitted through a 3 cm thick tissue sample. In terms of US, the used frequencies should be low enough to have good tissue penetration in centimeters, but, unfortunately, lower frequencies provide lower spatial resolution. The suitable frequency range is 0.5−5 MHz, and the US intensity should be less than the safety limit set by the U.S. Food and Drug Administration (FDA), which is 1.9 MI (mechanical index). US setups typically employ 2 MI for the focusing beam, which equals a 1.2 MPa pressure for 1 MHz US. The purpose of this paper is to review recent research into AOI development in terms of theoretical modeling and simulations as well as medical applications with a focus on research published since the last review in 2017 [10]. The Section 2 provides an overview of theoretical modeling strategies and numerical simulation methods, followed by Section 3, which explores recent applications of AOI in medicine. The Section 5 of this review offers concluding remarks and prospects for the field.

## 2. Modeling of Acousto−Optical Imaging

### 2.1. Acousto−Optic Interaction within Multiple Scattering Media

**Figure 1:**
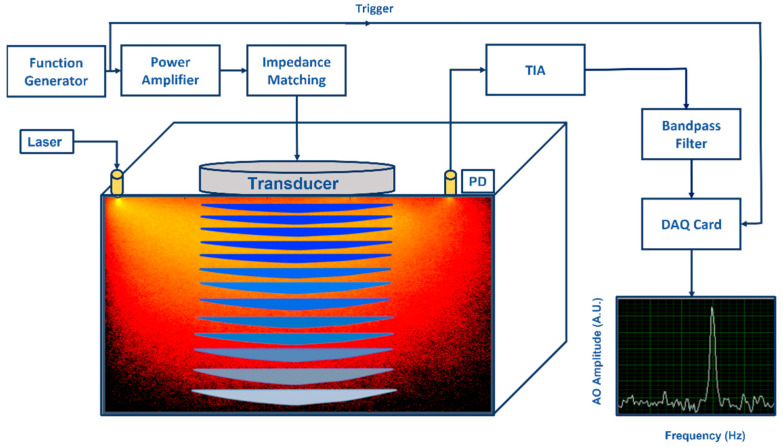
An illustration of an acousto−optic detection system: Acoustic part: Function generator generates amplitude modulated signal at fixed frequency (kHz) and power amplifier drives the ultrasonic transducer. Acoustic waves generated by the transducer propagate into the substance causing pressure changes in the substance. Optical part: Light illuminated by a laser propagates into the substance and a part of the diffused photons pass through the ultrasonic modulated areas causing light modulation to these photons due to the acousto−optic effect. A photo detector (PD) converts optical power to photocurrent (both DC and AC) and the transimpedance amplifier (TIA) converts it to voltage. When voltage signal is effectively filtered by a narrow bandpass filter at the fixed frequency, modulated light can be distinguished visible as a narrow power peak in frequency domain.

A biological tissue is a high scattering multilayer medium with heterogeneous physical properties [3,4,10,12]. Applying a US field to a tissue will change its optical properties in time and space, making light propagation in it more complicated. As the transmitted light is detected, a speckle pattern forms owing to interference of different phase differences. Figure 1 illustrates what happens when light frequency is modulated or “tagged” as it passes through the US focus region. As a result of this modulation, the speckle pattern is blurry and varies due to time−varying US, and the detected speckle spectrum contains *n* orders of sidebands. Additionally, the tagged signal is small compared to untagged light [3,13].

Several mechanisms can cause tagging of light in a medium [3,10,14]. The first is the displacement or oscillation of scatterers by US waves; scatterers oscillating at US amplitudes cause a variation in the optical path, resulting in phase variations in light. The mechanism can only be valid when the mean free path is far greater than the acoustic wavelength [10]. The second mechanism describes phase variation caused by a refractive index change between scattering events. It is worth mentioning that refraction of light occurs between two scattering events due to changes in the index of refraction. Because of this mechanism, optical path lengths, and therefore phases, are modulated. This, in turn, modulates the intensity of the resulting speckle pattern [14]. Lastly, the third mechanism is caused by variation in density and consequent changes in the optical properties of the medium due to US perturbation, including changes in the absorption coefficient, scattering coefficient, and index of refraction. According to previous studies, the first two mechanisms need a coherent light source, whereas the third mechanism is an incoherent phenomenon, in which the US modulated signal is very weak, so the third mechanism can be ignored in the case of a coherent light source.

In coherent light illumination, the relative influence of the first two mechanisms, ultrasonic−induced displacement of scatterers and ultrasonic−modulated index of refraction, depend on properties of the medium and the used waves. The relative influence of these two mechanisms is changed by the scattering coefficient and the wavelengths of the US and the probing light. The strength of both mechanisms is comparably high, but the ultrasonic−modulated index of refraction becomes dominant when the acoustic wavelength becomes larger than a critical fraction of the mean free path of the photons [14,15]. It is worth mentioning that the modulation depth is closely related to the intensity of the speckle patterns produced by US−induced variation of the optical phase of a coherent light. In studying biological tissues in vivo, the speckle decorrelation time is usually shorter than 1 ms [16] due to scatterers’ irregular (non−modulated) movements in the microfluidic system of living tissues. Hence, differing from tissue phantom studies, in vivo detection techniques must be fast enough to avoid speckle decorrelation.

On the other hand, US modulation of light can be explained by mixing two waves, US and optical waves. When interacting with light, US generates sidebands in scattered light that are shifted by multiple US frequencies. In practice, only the first two sidebands containing a number of photons or power spectra (+1 order of sidebands) are detected and processed [10,17]. In contrast to untagged photons that are not shifted in optical frequency when measured, tagged photons usually exhibit one US frequency shift, and their number is very low in comparison to the background of untagged photons, because deep tissues have a large diffuse volume, but very little US focus. Therefore, very efficient filtering techniques are necessary to remove as many untagged photons as possible before detection, or very sensitive detection techniques must be applied to detect very weak modulations.

### 2.2. Theoretical Modeling and Computational Simulation

A solid basis for the AO effect theory is provided by an electromagnetic wave propagation model in a material medium. In a general case, such a model is based on Maxwell equations for a dielectric whose permittivity is modulated by acoustic wave propagation [18]. In addition, phenomenological theories can be employed for multiplying scattered light using the radiative transport equation (RTE) or diffusion approximation (DA) to the RTE [19,20]. The theory of diffraction of light by an US wave was first proposed by Brillouin [21] in 1922 and then proved experimentally by Debye and Sears [22] as well as Lucas and Biquard in 1932. Raman and Nath proposed an analytical model of the AO effect—also called Raman–Nath diffraction—in a homogeneous non−absorbing and non−scattering medium [23]. A numerical simulation of time−reversed ultrasonically encoded optical focusing (TRUE) was developed by Jang et al. [24] to explore the penetration depth limit of TRUE optical focusing, considering the limitations of incident light fluence and TRUE’s recording time. They used diffusion approximation with a zero−boundary condition for light propagation into a US−focused region in deep tissue. US frequency−shifted light was determined using Raman–Nath theory, and the intensity of frequency−shifted light propagating back to the tissue surface was determined to calculate detection shot noise. In addition, they determined the relationship between shot noise and focus contrast (peak−to−background ratio, PBR) and came up with a practical depth limit of between 30 and 100 mm. It is worth mentioning that most of their assumptions and parameters are not reasonable in practical applications. Walther et al. [10] proposed a simple theoretical model based on the diffusion equation to evaluate the imaging depth of two interesting non−invasive imaging techniques, AO and PA. This model calculated absorption contrast levels, where a drop of one percent in blood oxygenation resulted in a decrease of 0.37 percent in the absorption coefficient (at the wavelength of 880 nm). For both techniques, limiting noise sources were identified, and assumptions were considered to evaluate general optical performance versus depth. It was found that the absorption contrast, and hence the oxygenation contrast that could be distinguished, was three orders of magnitude greater for AOI than for PAI at a depth of a few centimeters. They showed analytically that AOT with rare−earth−ion crystals as spectral hole−burning filters could be considered a deep non−invasive imaging technique superior to PA.

By utilizing the Monte Carlo (MC) simulation, light modulation in tissues can be precisely examined to gain an insight into the tagging mechanism. Wang [15] originally developed the acousto−optic Monte Carlo (AO−MC) model for homogeneous media to simulate US tagging of photons. AO−MC models were later developed for inhomogeneous and multiply scattered light [25]. Wang’s MC model has been the basis for several simulation−based studies, since the model can be used for simulating photon propagation and determining light’s phase change under continuous US perturbation in a non−absorbing homogeneous isotropic medium. The model was further extended to include a graphics processing unit (GPU) and a method to acquire the speckle pattern [26]. Additionally, the model was further refined for inhomogeneous media with designated US regions [27]. Gunther et al. [5] used MC simulations to analyze the contrast−to−noise ratio of AO tomography with slow light filters against possible imaging depths. They also studied the model’s ability to combine spectral hole burning (SHB) with AOI systems. To understand how much contrast can be achieved within a biological medium, they calculated the contrast−to−noise ratio (CNR) of both reflectance (for different source–detector distances) and transmittance configurations.

**Figure 2:**
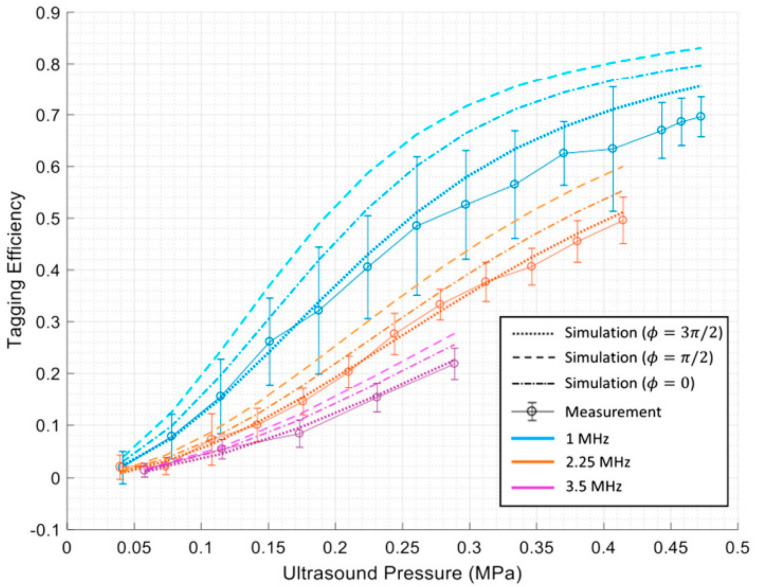
The results of Huang et al. [17] indicating tagging efficiency vs. US pressure and frequency.

Huang et al. [17] have conducted MC simulation studies to gain a deeper understanding of the interaction between US and light and the quantification of tagging efficiency. They used the MC method to simulate the interaction between the two coherent modulation mechanisms to determine overall tagging efficiency. They showed that by considering the higher orders of modulation in the measurements, a higher degree of tagging efficiency could be achieved. They proposed a theoretical approach for obtaining tagging efficiency as the power of all frequency−shifted light over the power of light passing through the US region, which is more a robust and appropriate method. This knowledge is essential for estimating a system’s signal−to−noise ratio (SNR) and for improving detection methods to enhance SNR in US−assisted optical imaging techniques. They showed that the two US modulation mechanisms, particle displacement and refractive index change, counteract each other in scattering media. In addition, they quantified tagging efficiency versus US pressure and frequency via simulations and experiments. Their results indicate that tagging efficiency increases as US pressure increases. In contrast, a higher US frequency leads to lower amounts of tagging efficiency [17]. Figure 2 shows their results.

Gunther et al. [28] developed a MC model using the CUDAMCML code, a MC model of steady−state light transport in multi−layered tissues based on NVIDIA’s Compute Unified Device Architecture (CUDA), to study the contrast−to−noise ratio (CNR) of AO tomography in biological tissue. Their results showed that when the imaging depth reaches ~5 cm in reflection mode or ~12 cm in transmission mode setups, the CNR exceeds 1.

Bocoum [29] et al. developed a new structured AO tomography method, which allows a partial recovery of resolution. For image reconstruction, they presented a generalized Fourier slice theorem and a generalized filtered back−projection formalism. Field−II open−source software was used to simulate the propagation of US pulses using software operation based on the far−field calculation method detailed in [30].

Hill et al. [31] presented a rigorous description for modelling the interaction between US and light for US pulses in nonlinear media under pressures ranging up to the medical safety limit. Their model simulations agree well with measurements conducted with fully characterized US pulses. Furthermore, their results demonstrate that movements of acoustically induced scatterers can be ignored during AOI modelling. This modelling approach is based on re−implementing, iterating, and finally showing that, under certain limitations, the work performed by Huang et al. [17] is practical for describing the tagging process in high−pressure US. They finally presented and validated a simulation package [32] for interaction between arbitrary optical and acoustic fields in scattering media.

Hsieh et al. [33] combined MC eXtreme (MCX) simulation software and intralipid−phantom experiments to investigate the use of a HIFU−induced heating tunnel to reduce photon scattering and enhance the delivery efficiency of light within biological tissues. They assessed the correlation between heating tunnel size, temperature change, and the fluence of light. Their results indicate that the delivery of light energy increases with rising temperature, reaching a maximum when the size of the tunnel slightly exceeds the width of the laser beam.

The angular spectrum method is a frequency domain numerical simulation technique applied to compute the propagation of US beams [34]. For practical details about the implementation of the angular spectrum method, please refer to [35]. Using this method, Adam et al. [36] developed a numerical model to calculate the acoustic field generated by the HIFU source. A finite−difference time−domain solution to Pennes’ bioheat equation was used to model the temperature field resulting from US absorption. An optical dose model based on measurements of tissue properties was used to calculate changes in tissue optical properties. This simulated acoustic field and the resulting effects on tissue properties were used to calculate phase modulations imparted on the optical field. Modeling of light propagation in the optical field was performed using an open−source GPU−accelerated MC algorithm that accounted for light−acoustic interactions and the detection of AO signals.

A Finite Element (FE)−based simulation method can also be used to solve AO effects. One of the first FE−based acousto−optic 3D simulation models, which also included a comparison with the MC method, was presented by Wang et al. in [37]. Their FE based−simulation results were close to those obtained with MC−based simulations, while requiring a more reasonable computational time. COMSOL Multiphysics is an FE application that incorporates light propagation, PA signal generation, and sound wave propagation in soft tissues. It provides the add−on modules “Wave Optics Module” and “Acoustics Module”, which can be combined to study these phenomena with 3D modelling [38]. Using COMSOL Multiphysics, Song et al. [39] simulated light propagation in biological tissue in a range of US fields, using the photon transfer equation and the “Coefficient form PDE” interface in the software. They also discussed the relationship between US−modulated scattering light and biological tissue optical properties. Acousto−optic signals exhibit exponential decay with an increase in the medium’s absorption and scattering coefficients, because the medium’s absorption coefficient influences the acousto−optic signal more than its scattering coefficient. Ling et al. [40] used COMSOL Multiphysics to investigate the relationship between the acoustic radiation force (ARF) and different types of acoustic pulses and waveforms to obtain optimum patterns for US excitation and pressure fields. Using their simulation results, they also conducted experiments on the enhancement effect of US generated ARF on diffuse correlation spectroscopy (DCS) data and blood flow measurements. It turns out that FE methods are faster and more flexible than MC, and they can measure photon density everywhere as well as boundary fluxes. However, they cannot deduce the history of individual photons [41].

Other alternatives for FE−based commercial simulation software are open−source Gmsh [42] and GetDP [43], which are often combined under the name ONELAB [44]. ONELAB is a FEM solver, which uses Gmsh for creating a FEM mesh, and GetDP for solving generic partial differential equations (PDEs) with the FEM method. Advantages of using Gmsh include its ability to create user−defined meshes, while also having standard interfaces with other commonly used mesh and computer−aided design (CAD) software such as STEP, IGES, and STL. Fadden et al. [45] used ONELAB for photo− and RF−acoustic computed tomography. To solve optical, electromagnetic, and acoustic propagation problems, ONELAB uses solutions to the optical diffusion equation, Maxwell’s equations in the frequency domain, and wave equation in the time domain. As shown by tests on a homogeneous phantom and an approximate breast phantom, ONELAB is an effective tool for both photo− and RF−acoustic simulations. It provides invaluable support for developing new reconstruction algorithms. Giuseppe et al. [46] studied US combined with DOT to increase imaging resolution for accurate lesion detection. They employed a self−generated breast 3D model, k−wave tool for US simulation, and machine learning for lesion classification. Eventually, the lesions were classified in the accuracy of 75%.

## 3. Experimental Biomedical Studies

To be successful as medical imaging technology, AOI must overcome several challenges. Due to Brownian motion and physiological motion, such as breathing, heartbeat, and blood flow, speed is an essential factor when performing AO measurements in biological tissue. The decorrelation time of light speckles is on the order of 1 ms [47]. By examining diffusing wave spectroscopy, Qureshi et al. [47] determined the relationship between decorrelation time and the depth of a point−like light source inside a living mouse brain. To detect tagged signals, more complicated detection methods are needed, which can sometimes fall outside the time constraints. One solution to overcome this limitation is offered by the off-axis heterodyne holography method [48,49,50,51]. Hussain et al. [52] showed that AO tomography in conjunction with heterodyne parallel speckle detection can be used to locally measure blood flow deep inside highly scattering media. According to the authors, the AO signal is sensitive to blood flow speed in the US focus area.

As biological tissues are highly optical scattering, it becomes infeasible to focus light with traditional lenses beyond approximately one transport mean free path (~1 mm in human skin). Liu et al. [53] developed a rapid ultrasonically modulated light setup, based on a high−speed TRUE focusing system, which can tolerate rapid speckle decorrelation on the scale of 5.6 ms. The setup successfully focused diffuse light in dynamic scattering media containing ear tissue from a living mouse, and imaged an absorptive target embedded between the mouse ear and a ground glass diffuser. Later, Ruan et al. [54] developed an integrated system with fast TRUE focusing and path clamp electrophysiology for simultaneous optogenetic stimulation and neural activity monitoring within living brain tissue ex vivo. Ruan’s system can focus light through 2 mm−thick living brain tissue at the 532 nm wavelength, and increase the spatial resolution of neuronal excitation by four times compared to conventional focusing at this wavelength. 

Furthermore, Liu et al. [48] studied the effect of human skull on ultrasonically modulated optical signals in vitro, by off−axis heterodyne holographic detection. A CW laser with a 35 mW output at the 671 nm wavelength was used as a light source with the light beam divided into reference and sample beams. The sample arm consisted of two human parietal skull bones with a thickness of 3~5 mm and a black plastic tape with a transmittance <0.1%. The skull bones were separated by 1.5 cm and the black plastic was attached on the internal surface of the first skull to simulate an absorber in the brain. A 1 MHz US transducer produced US passing through the first skull bone with a focal pressure amplitude of ~0.34 MPa. The sample and the reference beams formed an interference pattern on the recording camera, and the interferogram was then operated on by a two−dimensional Fourier transform to reconstruct the field of ultrasonically tagged light. By processing the intensity signal of this field, the black absorbing tape was successfully imaged in the lateral direction. Albeit preliminary, the result seems to validate the feasibility of using AO tomography to image the brain through the human skull.

### 3.1. AO Imaging of Tissue

AOI was introduced into biological imaging by Marks et al. who demonstrated the possibility of imaging homogeneous turbid media by detecting US modulated light using a single pin photodiode detector [55]. In a later study, Wang et al. used AO to obtain 2−dimensional images of objects in turbid media phantoms [56].

Jang et al. [57] proposed a new gating operation named space gating, based on selectively measuring ballistic waves to filter out multiply scattered waves. In this method, the image is reconstructed using acousto−optically modulated ballistic light at the object plane. Using space gating, multiply scattered waves are suppressed 10–100 times, which enabled the visualization of skeletal muscle fibers in whole−body zebrafish 30 days after fertilization. With the help of this technique, optical−resolution microscopy can achieve the ultimate imaging depth determined by the ballistic wave detection limit.

A computational imaging approach developed by Rosenfeld et al. [58] allows optical diffraction−limited imaging using conventional AOI. To achieve this, speckle correlations are analyzed in conventionally detected US−modulated scattered light fields to extract diffraction−limited imaging information. This strategy proposes the idea that since the estimation of the Fourier magnitude of the field within the acoustic focus area is possible through “memory−effect” speckle correlations, scanning the acoustic focus also provides a reliable diffraction−limited reconstruction of extended objects using ptychography (i.e., the US focus is exploited as the scanned spatial−gate probe needed for phase retrieval in ptychographic imaging). A 40−fold improvement in resolution over conventional AOIs was experimentally demonstrated by the authors.

In DOT, light is highly scattered within biological tissues. To reconstruct images, multiple detectors are placed along the surface of the medium to record where light exists. In many cases, optical properties cannot be appropriately extracted from these scattering medium measurements. By providing additional information, AOI can reduce the inadasasequacy of the reconstruction algorithms used in traditional optical tomography. Several research groups have developed algorithms for mapping these parameters [10], with further details available in [59,60,61,62,63,64,65,66,67,68,69,70]. Using algebraic inversion formulas, Chung et al. [71] obtained scattering and absorption coefficients through reconstruction with Lipschitz stability. They demonstrated that one can derive the scattering coefficient from boundary measurements of a one−parameter subset of illuminations and the absorption coefficient from boundary measurements of a single illumination. The stability of the reconstruction has also been studied by Chung et al. [72,73]. In their study, they examined the role of the Knudsen (Kn) number in AO image reconstruction and found that as Kn decreases, photons scatter more frequently and information is lost, resulting in an unreliable reconstruction. To reduce this issue, they posited that the laser beam must be highly concentrated and explicitly showed that this concentration is exponentially dependent on Kn. In a new study, Bocoum et al. [74] described a new approach for imaging acoustics using the spatio−temporal structure of long−period acoustic plane waves. This approach is particularly useful for detectors using long integration times. In their paper, they demonstrated how to reconstruct an image by measuring its two−dimensional Fourier components. A significant aspect of their research is that 2D Fourier Transform acousto−optic imaging (FT−AOI) relies on long−duration acoustic pulses, which may be suitable for imaging in vivo, because of their compatibility with camera−based detection setups. 

**Figure 3:**
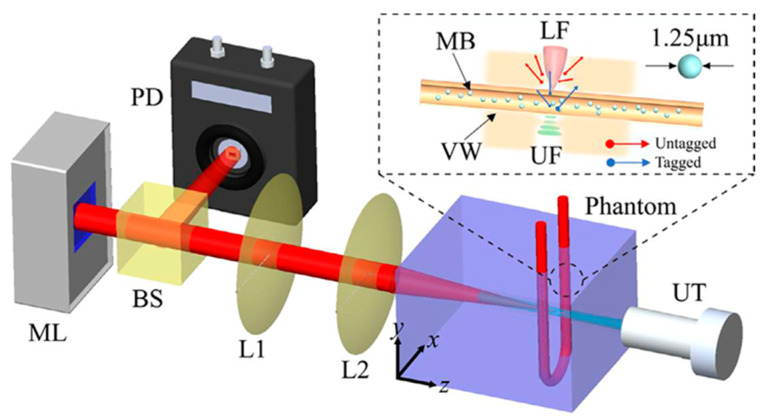
Illustration of the laser feedback AO Tomography system including microchip laser (ML), beam splitter (BS), PD, lenses (L1, L2,), and US transducer (UT), for imaging microbubbles (MB) inside vessel wall (VW). A reflective and autocollimation configuration is used for the whole system. The ML is pumped by laser diodes, which modulate the output power by reflecting backscattered photons. Light reflected from the BS reaches the PD and incident light reaches the front surface of the phantom. UT is placed coaxially with the laser beam, where the US focus (UF) overlaps the laser focus (LF). A function generator produces sinusoidal bursts at a repetition rate of 100 Hz, and is then amplified. Light and US are focused in the tube simultaneously. During UT operation, measuring light is shifted (3 MHz), whereas background signal is not shifted. Reprinted from [75], Copyright (2022), with permission from Elsevier.

By using microbubbles in US modulated laser feedback system in the reflective mode Ziyu et al. [75] showed that CNR increases from 0.78 to 3.73 at a penetration depth of 5.5 cm with 0.75 mm lateral resolution, compared to traditional AOI. Moreover, it was demonstrated that CNR increases with higher microbubble concentrations. The laser feedback AO Tomography system is shown in Figure 3. Moreover, Ahiad et al. [76] used silicon photodiodes (PD) instead of photomultiplier tubes (PMT) in their homodyne technique with micro bubbles and improved CNR four times. The setup may be used in both diagnostic and monitoring applications, such as deep vessel imaging.

### 3.2. Optical Fluence

Quantification in terms of determining local optical fluence in biotissue is of fundamental importance for biomedical optical measurements. Ultrasonically modulated scattered light has been used to noninvasively measure local optical fluence in optically inhomogeneous scattering media to correct photoacoustic signals [77,78,79]. The method is based on including local tagged photons using US modulation and the photon path reversibility principle. Measuring optical fluence in the depth direction and using the measured fluence to compensate for the raw PA signal amplitude, allows the fluence−compensated PA signal or the optical absorption of absorbers embedded in the tissue phantom to be corrected to an accuracy of 5%, even if the fluence variation exceeds one order of amplitude. Measuring fluence can eliminate optical fluence−related artifacts from PA imaging, or similar artifacts in other optical techniques, such as fluorescence imaging, DOT, and photodynamic therapy. An example of using combined PA imaging and ultrasonically modulated light to correct optical fluence involved a quantitative measurement of blood oxygen saturation in tissue phantoms [79], in which fluence compensation was not only performed spatially, but also at different wavelengths. Experimental results indicated that blood oxygen saturation values using AO−assisted fluence−compensated PAI were in good agreement with those measured by an oximeter, whereas values obtained by PAI alone showed a positive bias. Recently, the team applied combined PA−OA tomography to small−animal imaging to investigate fluence−corrected PA imaging in a single instrument. They demonstrated that correcting spatial and spectral fluence variations in PA images establishes a direct relation between image value and the optical absorption coefficients of chromophores, with a fluence correction accuracy of 8% [80]. However, there are two challenges for the in vivo application of the setup: one is speckle decorrelation caused by tissue dynamics and the second is instrumental complexity, due to the use of different types of lasers for AO and PA tomography. To solve these problems, the setup could use a pair of coherent pulse lasers with nanosecond pulse duration. This is because a pulsed laser can deliver sufficient light in a single pulse to form a speckle image, thereby shortening measurement time relative to tissue decorrelation time. Such a laser could be used to perform both PA and AO measurements, thereby reducing instrumental complexity.

The determination of light fluence (i.e., the optical energy delivered per unit area) distribution is required to reconstruct the true absorption coefficient spectrum for functional and quantitative PA imaging, especially at large depths [80,81]. For in vivo applications, AO is a useful method, since it does not require prior knowledge of the optical properties of the medium, works under various illumination conditions, and can be applied to different PA imaging geometries [80]. Hussain et al. [80] examined the feasibility and potential of fluence−corrected PA imaging embedded in a single instrument to image small animals. Experiments were conducted using phantoms, an ex vivo tissue sample, and freshly sacrificed mice. The authors demonstrated that correcting for spatial and spectral fluence variations in PA images improves the quantitative estimation of blood oxygen saturation by directly attributing image value to optical absorption. Bioluminescence imaging (BLI) in vivo has poor spatial resolution, because of strong tissue scattering, which also adversely impacts quantitative characterization accuracy. US modulation of emitted light is an effective solution to enhance spatial resolution as it reduces the effects of light scattering [82]. Ahmad et al. [82] utilized continuous US excitation at 3.5 MHz within a tissue−mimicking phantom, because it modulates the incoherent light emitted by the embedded bio− or chemiluminescent sources. Their results showed that these hybrid techniques have improved spatial resolution and yield more accurate quantitative data than traditional BLI techniques.

## 4. Medical Applications

**Figure 4:**
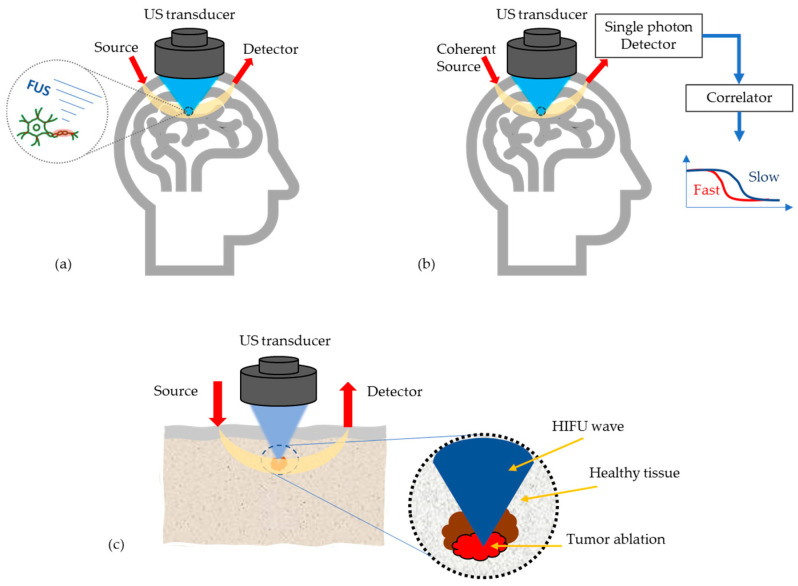
Most potential medical applications of AO techniques, (**a**) Monitoring high intensity focused US (HIFU) effects in transcranial applications, (**b**) Enhancement of the optics based cerebral blood flow and oxygenation measurements, (**c**) Tumor detection and its ablation monitoring.

At present, most research work on AO is still limited to simulations and tissue phantom studies, due to device limitations and/or the fact that the imaging/sensing time is longer than the decorrelation time of living biological tissue. However, there are few potential medical applications of AO emerging, see Figure 4, which will be discussed in the following.

### 4.1. Monitoring HIFU Effects in Transcranial Applications

HIFU is a non−invasive surgery method, in which a localized area of the body is heated rapidly, resulting in irreversible tissue necrosis caused by the high intensity. Due to patient and environment−dependent factors, a reliable treatment monitoring and guidance technique is imperative for this technique’s efficacy and clinical acceptance [36]. Currently, the only guidance methods used in clinical practice are diagnostic US and magnetic resonance (MR) imaging thermometry [36]. In addition to providing sufficient contrast between necrotic and healthy tissue, a suitable monitoring technique should also be cost−effective, uncomplicated, portable, and insensitive to patient movement [83]. In thermally damaged tissue, optical scattering and absorption coefficients increase due to the severance of phospholipid cellular membranes and the denaturation of intracellular and extracellular proteins [84]. In recent years, the applicability of AO imaging for the real−time monitoring of thermally induced damage in ex vivo tissues during HIFU exposure has been demonstrated. In AO imaging, the flux of phase−modulated light can be directly monitored to determine the effects of HIFU−induced heating in the focused region on the optical scattering and absorption properties of tissue [36]. By quantifying optical properties, research conducted by Adams et al. [85] demonstrated the capability of AOI in real−time monitoring of induced changes in chicken breast tissue (ex vivo) during HIFU ablation. They showed that HIFU−induced lesions can be detected by AO at varying depths depending on sensor geometry, wavelength, lesion volume, and tissue type. It is possible to detect breast cancer up to a depth of 50 mm and prostate cancer up to 25 mm [86]. Optical penetration depth decreases due to increased absorption and scattering during lesion formation. Using a modeling−based approach for the optimal design of a HIFU system with AO monitoring, Adams et al. [36] developed a treatment strategy for large volumes. They assessed the system’s robustness to changes in tissue thickness, lesion location, and lesion properties. They also showed that in the case of a single lesion, the effects on AO signals can be minimal, while HIFU therapy applications that cause multiple lesions, show a greater impact. Moreover, the orientation of the HIFU source and optical transducer could have a substantial impact on signal detectability. Based on spherical spectrophotometry, Raymond et al. [87] determined HIFU’s effect on scattering and absorption coefficients in isolated chicken breasts. Optical penetration proved greatest in NIR windows ranging from about 700 nm to 1100 nm. Compared to unexposed chicken breast tissue, the scattering coefficient of damaged tissue was 250% and the absorption coefficient 100% higher. Their proposed ex vivo study cannot evaluate the effect of blood circulation on optical parameters. Biological tissues exhibit significant light scattering, resulting in light defocusing and limited penetration during optical imaging and photothermal therapy in biomedical applications. Hsieh et al. [33] used high intensity−focused US (HIFU) to create a heating tunnel to reduce photon scattering and enhance the efficiency of light energy delivery. Results from their simulations and phantom experiments demonstrate that a HIFU−induced thermal effect improves light fluence by 3%.

### 4.2. Enhancement of the Optics based Cerebral Blood Flow and Oxygenation Measurements

Diffuse correlation spectroscopy (DCS) is an effective method for measuring cerebral blood flow (CBF) in many clinical settings. CBF measurements in adults are difficult due to DCS’ reduced sensitivity to blood flow changes at deeper tissue depths. By using acousto−optic modulated diffuse correlation spectroscopy (AOM−DCS) [88], which combines DCS sensitivity with US resolution, it is possible to improve the spatial resolution of the optical signal based on the region affected by US waves. Robinson et al. [88] developed a quantitative model for estimating perfusion based on AOI−DCS in the presence of US continuous waves (CW), which was supported by theoretical calculations, MC simulations, and experiments on phantoms and human subjects.

Caccioppola et al. investigated US−tagged near−infrared spectroscopy NIRS (UT−NIRS) to improve the capability of NIRS. They measured the cerebral flow index (CFI) of 40 subjects, half of whom were healthy, while the other half were brain dead. Their results demonstrate that UT−NIRS can detect a perfused brain in brain−dead patients in the absence of cerebral brain flow. They found that a true appraisal of cerebral perfusion may not be able to separate signals from extracranial structures [89]. Furthermore, Walther et al. [9] demonstrated that the AO technique based on a spectral hole burning filter is suitable for non−invasive optical imaging of oxygenation levels in frontal areas of the human myocardium.

### 4.3. Tumor Detection

AOI has been considered as a promising technique for tumor detection and was initially evaluated for breast tissue monitoring. The chicken breast tissue and multimodal phantom evaluations presented in [55,56,90] show AO’s capability to detect even small sized abnormalities which could be missed in mammography or in conventional US examination. However, one of the main challenges related to breast cancer detection is the large penetration depth requirements.

Acousto−optics is also shown capability to detect melanomas and its metastates in liver [91] Since melanin deposits in tumors, melanom metastates appear often as highly pigmented with a dark color which makes them easy to detect with AO. Laudereau et al., who presented the first US modulated optical images of ex vivo liver samples in [92], proved that AO is able to locate metastates in regions where US alone was not able to capture them. However, AO images alone are difficult to interpret and thus it is suggested as complementary detection technique. For instance, AO/US multimodal platform is proposed to increase the sensitivity and specificity in tumor location and characterization [92]. 

As an example case is brought the uveal melanoma which is a common ocular tumor producing liver metastases with high probability [93]. The amount of metastates in the liver effect on the treatment plan: patients with few metastates may be treated surgically whereas numerous metastates may require, e.g., chemotherapy. Therefore, non−invasive AO technique would facilitate treatment plan as it may allow more precise estimation of the metastates [92].

In Table 1, a summary of selected AO studies published between 2018–2022 with a short explanation of the study purpose, key results and their possible medical application is presented.

## 5. Conclusions

AOI was first presented more than twenty−five years ago as a hybrid technique to improve the spatial resolution of diffuse optical imaging to the order of millimeters at centimeters depth in tissue−mimicking phantoms. AOI is based on detecting tagged photons or modulated light from a tissue’s US focus region and sensing optical changes there. To that end, it removes the effect of photons or light from all other locations (background), resulting in improved spatial resolution. In many AOI applications, the US transducer focuses on a small region in a tissue at a depth ranging from one to a few centimeters; photons are only tagged within this focus area. Because only a small part of incident light passes diffusely through the US focus, and as only a portion of the photons are tagged, a relatively small number of tagged photons is diffusely reflected from the tissue. Therefore, the SNR and detection sensitivity are much smaller than in diffusion based optical sensing. Many AOI simulations have been performed using MC techniques, including simple and complex geometry, linear and nonlinear approaches, homogeneous and heterogeneous media as well as steady−state, time−resolved and frequency−domain procedures. Parallel computing has been applied to speed up the processes, together with more realistic mesh−based elastic light scattering models. MC simulations [17] have also been used to quantify tagging efficiency versus US pressure and frequency along with the contrast−to−noise ratio (CNR) determination of AOI [28]. In addition, a simulation package, presented and validated by [31], investigated AO with US pulses in nonlinear media at pressures reaching up to the medical safety limit to simulate interaction between arbitrary optical and acoustic fields in scattering media [34]. MCX based on parallel computing [33] was applied to investigating a HIFU−induced heating tunnel to reduce photon scattering and the results indicate enhanced light delivery efficiency within biological tissues. COMSOL multi−physics, based on the FE method, were used for AO modeling in [37,38,39,40]. Even though they are faster and more flexible than MC methods, FE methods cannot deduce photon histories.

Huang et al. [17] recently reported 70% tagging efficiency in a scattering phantom. However, this high tagging efficiency was achieved in an ideal case (both incident light and US overlapped in a small scattering sample, and all orders of tagged photons were taken into account), rather than in practice with incident light and focus US passing through a large scattering medium, where tagged photons are produced deep in the medium and only a small part of the tagged photons are reflected back to its surface. A photodetector accepts both tagged and untagged photons. Therefore, practical tagging efficiency, defined as the number ratio of tagged and untagged photons at the photodetector, is much less than that achieved by Huang. In addition, tagged photons are modulated in multiple orders, and detectors are only capable of recording first−order modulation. Considering these factors, we estimate that the modulation depth of detected signals should be on the order of 10^−4^ when the US focus is at a depth of a few centimeters within a tissue phantom. In effect, the deeper the US focus in the phantom, the lower the modulation depth of the signal. 

At present, most research work on AOI is still limited to simulations and tissue phantom studies. Probably most potential biomedical applications of AO are in near future supportive techniques that are combined with purely acoustics and optics−based modalities to obtain more quantitative information for clinical studies. For example, measuring fluence can eliminate optical fluence−related artifacts from PA imaging, fluorescence imaging, and photodynamic therapy. Combined with diffused correlation spectroscopy, fluence measurements can improve the spatial resolution of blood flow in deep tissue. 

Although PAI is entering clinical application, it still faces a great challenge, particularly in terms of human brain imaging. When photoacoustic waves from the brain propagate out of an adult human skull, they are strongly attenuated and distorted by wave mode transformation and unmatched acoustic impedance between the skull bone and soft tissues within the head. Since AO detects optical signals, optical parameter unmatching between bone and soft tissue is much smaller than acoustic parameter unmatching. This serves to greatly reduce the effect the skull has on the signals. Accordingly, we believe AOI is a better option to successful imaging and sensing of the adult brain. Furthermore, the imaging depth of AOI can reach 10 cm, about twice the depth achieved by current PAT [9].

## Figures and Tables

**Table 1:** A summary of selected AO studies published between 2018–2022.

Publication	Year	Purpose of the Study	Key Results	Type of Sample	Possible Medical Application
[52]	2018	AOI for locally sensing and imaging blood flow deep in highly scattering medium	AO signal relation to the speed of blood flow.	Phantom	Imaging blood flow in tissue
[89]	2018	Measuring cerebral flow index (CFI)	in brain−dead patients, that CBF is lacking, the UT−NIRS can indicate an apparently perfused brain.	Human brain	Measuring cerebral flow index (CFI)
[87]	2018	HIFU monitoring	During HIFU lesion, both absorption and scattering increased, resulting in a decreased optical penetration depth And AO signal.	Phantom	Real−time HIFU therapy monitoring
[80]	2018	PA imaging correction	In PAI, spatial and spectral variations in fluence can be corrected by AOI.	Phantom and Mouse	Quantitative estimation of blood oxygen saturation
[88]	2020	Enhance DCS spatial resolution	The AO−based blood flow induced scatterer dynamics is identical well (< 1%) with DCS−based measurement.	Phantom	Estimation BFI
[74]	2020	2D Fourier Transform acousto−optic imaging (FT−AOI)	The overall acquisition time in used photorefractive detection scheme is compatible with medical monitoring applications.	Phantom	In vivo imaging for functional or metabolism
[57]	2020	Extend the imaging depth of high−resolution optical microscopy	The space gating suppresses the multiply scattered wave by 10–100 times in a highly scattering medium.	Phantom	Deep tissue optical imaging for tumor and other tissue abnormality
[48]	2020	AO imaging through human skull	Possibility of imaging objects in tissue phantom through human skull sample.	Phantom	Human brain imaging noninvasively through the skull
[40]	2020	ARF improved DCS	ARF generated by US enhanced DCS data and blood flow measurements,	Phantom	Measuring blood flow
[17]	2020	Calculation of tagging efficiency and relationship between ultrasound optical modulation mechanisms	All orders of tagging efficiency in US focus can reach 70%	Simulation and phantom	Estimating maximal AO signal in tissue
[58]	2021	Improving AOI resolution for microscopic investigation.	40−fold improvement in resolution over conventional AOIs.	Phantom	Microscopy imaging
[51]	2021	Fourier transform acousto−optic imaging	2D FT imaging compatible with in vivo imaging, deep tissue imaging.	Phantom and simulation	Deep tumor and metabolism imaging
[75]	2022	Microbubbles effect in AOI	Using microbubbles, the contrast−to−noise is increased from 0.78 to 3.73 with the lateral imaging resolution of 0.75 mm.	Phantom	Deep vessel imaging
[76]	2022	Simplifying and reducing cost of AOI setup	By using a silicon PD, achieving over a 4−fold improvement in SNR in comparison to a PMT−based setups.	Phantom	Hand−on low cost AOI for skin imaging
[46]	2022	Utilize ultrasound wave in diffuse optical tomography simulation in order to increase resolution of the imaging	Accurate lesion detection, the accuracy of the classification of lesions was 75%.	Simulation	Discriminate malignant lesions from benign one

## Data Availability

Not applicable.
